# Long-Term Bacterial Dynamics in a Full-Scale Drinking Water Distribution System

**DOI:** 10.1371/journal.pone.0164445

**Published:** 2016-10-28

**Authors:** E. I. Prest, D. G. Weissbrodt, F. Hammes, M. C. M. van Loosdrecht, J. S. Vrouwenvelder

**Affiliations:** 1 Department of Biotechnology, Faculty of Applied Sciences, Delft University of Technology, Van der Maasweg 9, 2629 HZ Delft, The Netherlands; 2 Center for Microbial Communities, Department of Chemistry and Bioscience, Aalborg University, Fredrik Bajers Vej 7, 9220 Aalborg, Denmark; 3 Eawag, Swiss Federal Institute of Aquatic Science and Technology, Ueberlandstrasse 133, CH-8600 Dübendorf, Switzerland; 4 King Abdullah University of Science and Technology (KAUST), Water Desalination and Reuse Center (WDRC), Division of Biological and Environmental Science and Engineering (BESE), Thuwal 23955–6900, Saudi Arabia; 5 Wetsus, European Centre of Excellence for Sustainable Water Technology, Oostergoweg 9, 8911 MA Leeuwarden, The Netherlands; Universidade Federal do Estado do Rio de Janeiro, BRAZIL

## Abstract

Large seasonal variations in microbial drinking water quality can occur in distribution networks, but are often not taken into account when evaluating results from short-term water sampling campaigns. Temporal dynamics in bacterial community characteristics were investigated during a two-year drinking water monitoring campaign in a full-scale distribution system operating without detectable disinfectant residual. A total of 368 water samples were collected on a biweekly basis at the water treatment plant (WTP) effluent and at one fixed location in the drinking water distribution network (NET). The samples were analysed for heterotrophic plate counts (HPC), *Aeromonas* plate counts, adenosine-tri-phosphate (ATP) concentrations, and flow cytometric (FCM) total and intact cell counts (TCC, ICC), water temperature, pH, conductivity, total organic carbon (TOC) and assimilable organic carbon (AOC). Multivariate analysis of the large dataset was performed to explore correlative trends between microbial and environmental parameters. The WTP effluent displayed considerable seasonal variations in TCC (from 90 × 10^3^ cells mL^-1^ in winter time up to 455 × 10^3^ cells mL^-1^ in summer time) and in bacterial ATP concentrations (<1–3.6 ng L^-1^), which were congruent with water temperature variations. These fluctuations were not detected with HPC and *Aeromonas* counts. The water in the network was predominantly influenced by the characteristics of the WTP effluent. The increase in ICC between the WTP effluent and the network sampling location was small (34 × 10^3^ cells mL^-1^ on average) compared to seasonal fluctuations in ICC in the WTP effluent. Interestingly, the extent of bacterial growth in the NET was inversely correlated to AOC concentrations in the WTP effluent (Pearson’s correlation factor r = -0.35), and positively correlated with water temperature (r = 0.49). Collecting a large dataset at high frequency over a two year period enabled the characterization of previously undocumented seasonal dynamics in the distribution network. Moreover, high-resolution FCM data enabled prediction of bacterial cell concentrations at specific water temperatures and time of year. The study highlights the need to systematically assess temporal fluctuations in parallel to spatial dynamics for individual drinking water distribution systems.

## Introduction

Drinking water distribution can lead to aesthetic water quality deterioration, such as bad taste, malodourous or coloured water, and to operational problems such as bio-corrosion and/or fouling of water installations. Water quality changes are usually attributed to microbial processes in distribution pipelines, including growth of benign autochthonous bacteria and/or re-suspension or detachment of bacterial cells from pipe wall sediments and/or biofilms into the bulk water. Though the involved organisms are not necessarily hygienically relevant, water quality deterioration is of concern for water utilities, as it is a major cause of customer complaints and maintenance costs [1,2]. Numerous studies have shown that drinking water distribution networks are dynamic systems in which spatial and temporal changes take place in the autochthonous microbial community, i.e. in the type, amounts and proportions of bacteria present in water, as well as their activity and viability states [3–13].

Earlier studies of drinking water microbiology were predominantly based on HPC measurements [10,11]. However, this approach has been shown to be inappropriate for the enumeration of autochthonous bacterial communities, as only a minute fraction of drinking water bacteria are able to grow on conventional cultivation media [14]. Flow cytometry (FCM) has been proposed as an alternative microbial monitoring method for rapid enumeration of the total number of bacterial cells, evaluation of cell viability, and fingerprinting of bacterial communities in water samples [15–17]. Adenosine-tri-phosphate (ATP) measurement has also been suggested as a useful method to evaluate the viability of bulk water bacteria [13], particularly complementary to FCM [5,16,18–20].

Spatial and temporal microbial dynamics during drinking water treatment and distribution are likely affected by specific characteristics of each and every system, including raw water characteristics (chemical composition, organic and inorganic nutrients), treatment design and efficiency [4,6,15], residual disinfection implementation (or not) and disinfectant type [9,21], water temperature ranges [9,13], distribution pipeline materials and age [22,23] and residence times [6,10,21]. Fluctuations in raw water quality, treatment efficiency, and water temperature especially are likely to promote seasonal changes in the drinking water chemical composition (organic carbon, inorganic compounds, pH, …) and in microbial community characteristics after treatment and within the distribution system. For example, significantly higher ATP concentrations were observed in summer/autumn periods than in winter in a full-scale distribution system in the Netherlands [13]. Similar conclusions were drawn based on coliform growth [9] or HPC [10] in various drinking water distribution systems, while Pinto et al. [6] highlighted seasonal cycling in the taxa constituting the bacterial community in a drinking water distribution system in USA.

Even though seasonal changes in drinking water microbial quality in distribution systems have been highlighted before, temporal dynamics are poorly described and understood. Besides, seasonal variations are rarely taken into account in the design of studies addressing microbial dynamics in drinking water systems, as studies were generally performed at low water sampling frequency with poor or no investigation of temporal dynamics [12,13,24,25]. Moreover, only a few long-term microbial monitoring campaigns in drinking water distribution systems have been performed with alternative methods such as FCM and/or ATP and the techniques were generally not linked with abiotic parameters [6,26,27]. To date, there is no study available on long-term and detailed investigation of bacterial dynamics in full-scale drinking water distribution systems using a combination of conventional and alternative drinking water quality parameters.

In this study, temporal dynamics in water quality in a full-scale system distributing drinking water without detectable disinfectant residual was specifically studied by monitoring (i) a wide range of conventional microbial, organic, inorganic, and environmental parameters together with ATP and FCM (ii) over a 2-year period with (iii) high frequency sampling on a biweekly basis. The studied system is representative for water utilities treating surface water with extensive processes including ozonation and biological filtration, and distributing water without detectable disinfectant residual. The objectives of this study were (i) to assess long-term temporal variations in the bacterial community abundance and viability at the outlet of the drinking water treatment plant and at one location in the distribution system, (ii) to relate the microbial changes with variations in environmental factors possibly responsible for these variations, and (iii) to develop strategies for dedicated water quality monitoring programs.

## Materials and Methods

Ethics statement: Permission was obtained from the local water utility (Evides Waterbedrijf) to sample drinking water at the two studied locations. The field studies did not involve endangered or protected species.

### Study location

Drinking water sampling was performed at a Dutch full-scale drinking water treatment plant (Rotterdam area, The Netherlands) and in the corresponding distribution system. The study location was selected for its representativeness for full-scale surface water treatment and well-maintained distribution networks in industrialised countries, and for the availability of earlier datasets on the system [28]. The annual drinking water production was 40 x 10^6^ m^3^ per year. Surface water was treated at this location by coagulation, flocculation and sedimentation followed by ozonation, dual medium filtration, and granular active carbon filtration. Final disinfection (0.1 mg L^-1^ chlorine dioxide) was applied at the inlet of a large storage reservoir where the water was collected before distribution. The concentration of chlorine dioxide in the reservoir effluent was below the minimum detection limit of the analytical method (<0.001 mg L^-1^). The water was thus distributed in a network operating without detectable residual disinfectant.

### Water sampling

Drinking water samples were taken at the outlet of the water treatment plant (WTP) and at one location in the distribution network (NET) for the study of long-term temporal bacterial dynamics at both locations. The NET sampling site was selected based on an earlier study that showed significant differences in the water characteristics between this location and the WTP in the summer period [28]. Water samples were collected at the two locations every 2 weeks for a total period of 2 years. On every sampling day, 4 water samples were taken at each location at specific times and at 30 minutes intervals (09:00, 09:30, 10:00 and 10:30), in order to be able to identify potential outliers and unexpected events. In total 368 water samples were taken, i.e. 184 samples at the WTP effluent and 184 samples at the NET location. For practical reasons, the analyses of one or more parameters were not performed for a limited number of samples. The relative standard error on the flow cytometric results of the four samples taken on the same day was less than 15% in the majority of sampling days, and outliers were removed when this value was exceeded. The residence time of the water in the distribution system at the NET location is approximately 2 days, but this was not taken into account in the sampling scheme, similarly to the sampling strategy applied in our preliminary study [28]. Available methods to estimate residence time provide only rough estimations, and sampling of the same water fraction at both locations could actually not be accurately achieved.

The water sampling taps at both locations were running continuously during the entire 2-year study period. On each sampling day and time, the water temperature was measured and water was collected in separate bottles for each parameter to be measured, i.e. FCM total and intact cell concentrations and fluorescence fingerprints, adenosine tri-phosphate concentration (ATP), heterotrophic plate counts (HPC), total organic carbon (TOC), pH and conductivity. For comparison to other microbial parameters, *Aeromonas* counts were measured during the last 6 sampling months, since it is commonly analysed in the Netherlands as an indicator for bacterial growth in distribution networks. Additionally, the water utility provided data on concentrations of assimilable organic carbon (AOC) measured during the two-year period in the WTP effluent on independent sampling days than for the other parameters.

High-density polyethylene (HD-PE) plastic bottles (Identipack BV, the Netherlands) containing 2 mL L^-1^ of a mixed solution of sodium thiosulfate (20 g L^-1^) and of nitrilotriacetic acid (25 g L^-1^) were used to collect water for microbial analysis of ATP and HPC, and for FCM analysis, as routinely used by accredited laboratories for drinking water analysis in the Netherlands. Drinking water samples were collected in polyethylene terephthalate (PET) bottles without headspace (Identipack BV, the Netherlands) for pH and conductivity analysis and in glass bottles (Identipack BV, the Netherlands) containing sulphuric acid (8 mol L^-1^, 0.2 mL in 100 mL bottle) for TOC analysis. The water samples were transported on ice, stored at 4°C until analysis, and processed within 6 h after sampling.

### Conventional parameters (HPC, ATP, Aeromonas, AOC, TOC, pH, and electrical conductivity)

HPC was measured by Aqualab Zuid (Werkendam, NL), according to the Dutch standard procedure (NEN-EN-ISO 6222, 1999). In short, 2 mL of the water sample were transferred to a sterile Petri-dish and mixed with 20 mL of yeast extract agar. The agar was kept at 44°C before plating. The samples were incubated at 22°C for 3 days. ATP was measured by Het Waterlaboratorium (Haarlem, NL), as described previously by Magic-Knevez and Van der Kooij [29]. The ATP measurement is based on the emission of light resulting from the reaction between the ATP molecule and a luciferin/luciferase reagent (LuminATE, Celsis). For total ATP determination, ATP was first released from suspended microbial cells with nucleotide-releasing buffer (LuminEX, Celsis), while this step was not performed for the assessment of free ATP. The intensity of the emitted light was measured using a luminometer (Celsis AdvanceTM) that was calibrated with solutions of free ATP (Celsis) in autoclaved tap water following the procedure given by the reagent manufacturer. The detection limit of the method was 1 ng ATP L^-1^. Bacterial ATP concentrations were calculated by subtracting free ATP from total ATP concentrations. *Aeromonas*, AOC, TOC, pH and conductivity were measured by Aqualab Zuid (Werkendam, NL), according to Dutch standard procedures. TOC was measured on a Shimadzu TOC analyser (type TOC-V CPN). *Aeromonas* measurements were performed by filtration (0.45 μm pore-size, cellulose nitrate filter) of 100 mL of water samples and plate incubation at 30°C on a selective Ampicillin-Dextrin agar (Dutch guideline NEN 6263). AOC concentrations were determined following the procedure proposed by Van der Kooij [30] and according to the Dutch guideline NEN 6271. In short, pure bacterial strains P17 and NOX were inoculated in pasteurized water samples which were incubated in AOC-free glassware at 15°C. Bacterial growth was assessed by plate counting on Lab-Lemco agar incubated for 2 days at 25°C. The maximal growth was converted to organic carbon concentrations using specific yield values of the used organisms.

### Flow cytometry (FCM)

The protocol described by Prest et al. [17,28] was applied for water sample staining, FCM measurements and data analysis. Samples (500 μL) were pre-heated to 35°C for 5 minutes, stained with fluorescent dyes and incubated in the dark for 10 minutes at 35°C before measurement. For the determination of total bacterial cell concentrations (TCC), the samples were stained with 10 μL mL^-1^ of SYBR® Green I (1:100 dilution in DMSO; Molecular Probes), while 10 μL mL^-1^ of a working solution containing a mixture of SYBR® Green I (1:100 dilution in DMSO; Molecular Probes) and propidium iodide (0.3 mM) was used for the assessment of intact bacterial cell concentrations (ICC). FCM measurements were performed using a BD Accuri C6® flow cytometer (BD Accuri cytometers, Belgium) equipped with a 50 mW laser emitting at a fixed wavelength of 488 nm. The FCM is equipped with volumetric counting hardware, calibrated to measure the number of particles in a 50 μL volume fraction of a 500 μL sample. Measurements were performed at a pre-set flow rate of 35 μL min^-1^. A threshold value of 700 a.u. was applied on the green fluorescence channel (FL1). Bacterial signals were selected and distinguished from inorganic particles and instrument background on the BD Accuri CFlow® software using electronic gating on density plots of green fluorescence (FL1; 533 nm), and red fluorescence (FL3; >670 nm) [17]. FCM fluorescence “fingerprints” of the bacterial community were visualized as green fluorescence histograms [17]. The analysis of fingerprints is detailed in S1 Text.

### Multivariate numerical analyses of correlative trends

Computational multivariate analyses were conducted in R with dedicated packages [31] according to the numerical ecology framework proposed by Weissbrodt et al. [32] and applied by Besmer et al. [7], following Borcard et al. [33].

The simplified numerical methodology adapted here comprised the following sequential steps in a pragmatic computational approach (S2 Text): (*i*) concatenation of all microbial and environmental datasets in one single squared matrix with consideration of same measurement dates and/or using linear interpolation to fill in gaps in temporal datasets, and importation in R; (*ii*) panel plots of dynamics in individual measured variables over time in order to delineate global trends; (*iii*) pair-wise x-y plots between all measured variables for preliminary visual observation of apparent linear and monotonic correlations; (*iv*) self-contained correlation matrices following Pearson’s and Spearman’s approaches for delineating linear and monotonic correlative trends between variables, respectively; (*v*) normalization and standardization of the datasets of different dimensions (FCM and environmental variables) by scaling into zero mean and unit variance using the “decostand” function in R; (*vi*) separate computation of Pearson’s correlation and Spearman’s rank-order correlation coefficients between all variables, complemented by p-values at 95% confidence level in order to assess the significance of correlations; (*vii*) graphing and hierarchical clustering using the Ward algorithm of measured variables displaying analogous correlation patterns with all other variables in heat maps. This step enables a straightforward representation of the gradients in positive and inverse Pearson’s correlations and Spearman’s rank-order correlations between parameters and rapid identification of major correlative trends. Unless stated otherwise, Pearson and Spearman’s rank-order correlations were similar, and therefore only the Pearson’s correlations factors were used to describe results in the text and figures. Pearson’s correlation factors were chosen based on visual inspection of trends between the main parameters described in the results section, as shown in S2 Text. Heat maps of Spearman’s rank-order correlations are shown in S1 and S5 Figs.

This numerical methodology was applied on datasets collected from the effluent of the WTP and from the sampling location in the NET. In the latter case, the changes in microbial parameters (ΔNET) between the WTP and NET were considered for multivariate numerical computation.

## Results

### Variations in microbial parameters at two locations over a two-year period

**Fig 1:**
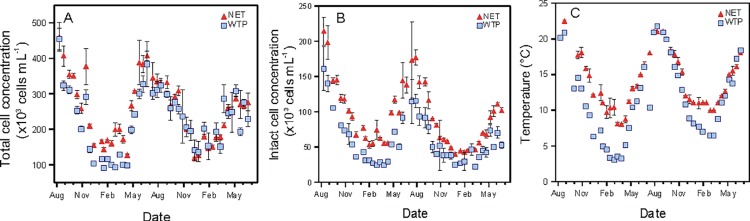
**Variations in time over two years (August 2012—June 2014) of (A) total bacterial cell concentration, (B) intact bacterial cell concentration and (C) water temperature at the water treatment plant (WTP) outlet and at one location in the network (NET).** Error bars indicate the standard deviation on four samples taken at the same location over a 2 h period.

**Fig 2:**
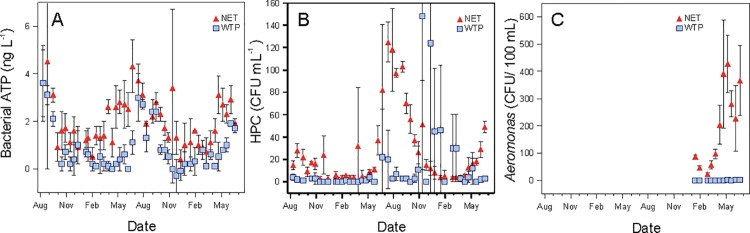
**Variations in time over two years (August 2012—June 2014) of (A) bacterial adenosine tri-phosphate (ATP), (B) heterotrophic plate counts (HPC), and (C) *Aeromonas* at the water treatment plant (WTP) outlet and at one location in the network (NET).** *Aeromonas* was measured starting from January 2014, i.e. during the last 6 months of the investigation period. Error bars indicate the standard deviation on four samples taken at the same location over a 2 h period.

Clear and reproducible seasonal trends were observed in the microbial characteristics of the water treatment plant (WTP) effluent, which in turn determined the characteristics of the water in the distribution network (NET) (Figs 1 and 2). Large seasonal variations in total and intact bacterial cell concentrations (TCC and ICC) were measured at the WTP effluent (Fig 1). The highest concentrations were recorded in the summer periods concurrent with the highest water temperatures, and inverse trends were observed in winter periods. The TCC ranged from 90 × 10^3^ to 455 × 10^3^ cells mL^-1^, of which only 26 ± 8% were intact. Seasonal variations were also recorded in bacterial ATP concentrations in the WTP effluent (ranging between <1 and 3.6 ng L^-1^; Fig 2). However, no trend was observed in heterotrophic plate counts (HPC), which remained below 5 CFU mL^-1^, except for 32 seemingly random measurements (out of 176 data points). *Aeromonas*, commonly analysed in the Netherlands as a bacterial growth indicator, was measured in the last 6 months of the study (January–June 2014) that covered a wide range of water temperatures (6.5–18.5°C). Similarly to HPC, the *Aeromonas* counts remained below 2 CFU 100 mL^-1^ in the WTP effluent. Low values for the percentage ICC, HPC and *Aeromonas* are attributed to the addition of chlorine dioxide before the storage reservoir at the end of the treatment train, although disinfectant residuals were not detectable after the reservoir.

Only minor changes in the drinking water microbial parameters were recorded at the NET location compared to the WTP effluent (Figs 1 and 2). A change in TCC could be observed between the two locations over the two-year study period, which was significant (P<0.0001 based on a paired t-test) but not systematic and relatively small (22% increase on average over the two years). However, increased ICC values (43 × 10^3^ to 214 × 10^3^ cells mL^-1^) and bacterial ATP concentrations (<1 to 4.5 ng L^-1^) were consistently recorded (Figs 1B and 2A), with a significant (P<0.0001) and clear increase in ICC (71% on average) in comparison to the TCC increase. Changes in FCM fluorescence fingerprints based on ICC were also detected between the two locations, which indicates a shift in composition of the intact bacterial community (Prest et al., 2013; S1 Text). Similarly, *Aeromonas* counts increased between the two locations (from 22 to 428 CFU 100 mL^-1^) along the 6 months monitored (Fig 2C), with the highest values being recorded at warmest water temperatures (15–18°C). An increase in HPC values between the two locations was only detected in the summer periods (May—October of each year, Fig 2B), with values reaching up to 125 CFU mL^-1^. Overall, the data shows that the major seasonal changes in TCC and ICC detected in the NET result from changes at the WTP, but that minor but detectable bacterial growth occurred during water distribution.

### WTP effluent characterization

**Fig 3:**
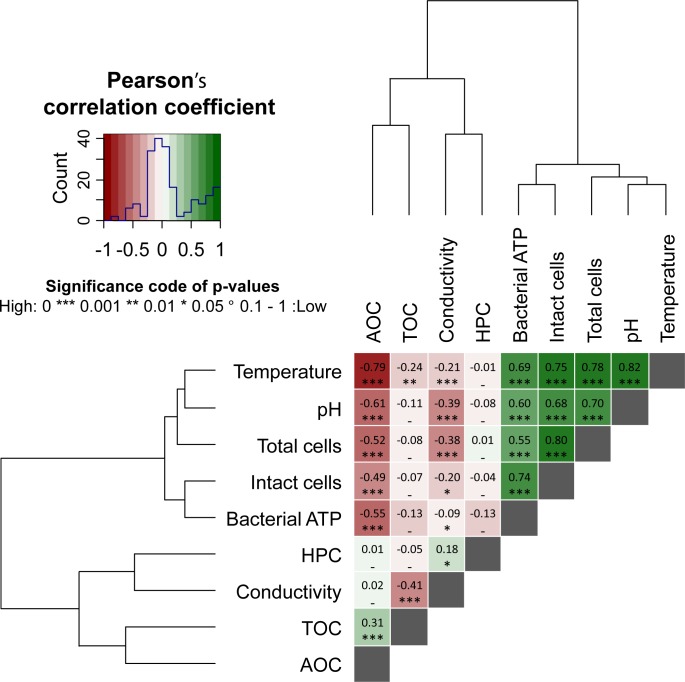
Heatmap of pair-wise Pearson’s correlation coefficients (*i*.*e*. linear trends) computed between the microbial parameters (namely total cell concentration, intact cell concentration, bacterial ATP, and heterotrophic plate counts (HPC)), and the environmental parameters (namely temperature, pH, electrical conductivity, total organic carbon (TOC), and assimilable organic carbon (AOC)) measured in the effluent of the water treatment plant (2-year dataset, 184 water samples in total). Hierarchical clustering using the Ward’s algorithm was first applied to reorder all parameters in clusters according to their correlation patterns as displayed by the dendrograms. The values and directions of the correlation coefficients are displayed according to the color key, *i*.*e*. positive correlations as green gradients from 0 to 1 and inverse correlations as red gradients from 0 to −1. Similar analysis of monotonic trends (*i*.*e*. Spearman’s rank-order correlations) between microbial and environmental parameters is available in S1 Fig. This approach is analogous to the one developed and used in [7] and [32].

Multivariate numerical analysis of the WTP effluent dataset suggested that water temperature had a strong linear relationship with microbial characteristics (Fig 3). A significant, strong, and positive linear correlation was found between water temperature and TCC (p<0.001; Pearson’s correlation coefficient r = 0.78; N = 176), ICC (p<0.001; r = 0.75; N = 173) and bacterial ATP concentrations (p<0.001; r = 0.69; N = 175). The linear correlation between water temperature and HPC was not significant, though a weak but significant positive monotonic correlation (p<0.001; r = 0.37; N = 175) was found between the two parameters using a Spearman’s test, which suggests a stronger influence of water temperature on HPC at higher temperatures (S1 Fig). This weak correlation is probably due to the fact that extremely low HPC values were found in the WTP effluent (82% of the HPC values were below 5 CFU mL^-1^).

**Fig 4:**
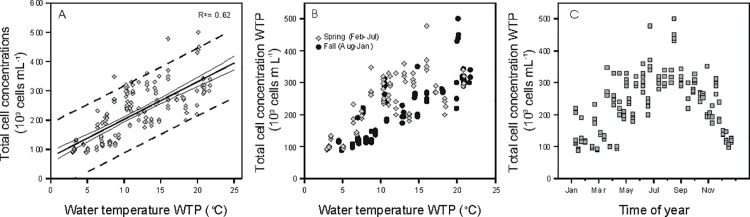
Relationship between water temperature and total bacterial cell concentration in the samples collected from the water treatment plant (WTP) effluent over the two years of investigation (August 2012 –June 2014). (A) 95% confidence interval (thin lines) and 95% prediction interval (dotted lines). (B) Discrimination between spring and fall samples. (C) Classification of water samples based on sampling time of year.

**Fig 5:**
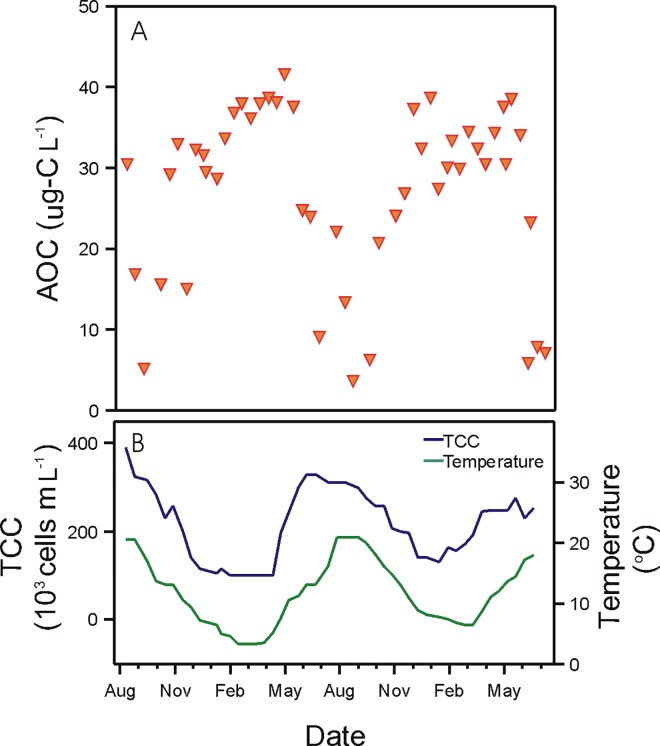
**Variations in time over two years (August 2012—June 2014) of (A) assimilable organic carbon (AOC) in the water treatment plant (WTP) effluent, compared to (B) variations in total bacterial cell concentration (TCC) and water temperature in the WTP effluent (data presented in Fig 1).**

The strong linear correlation between TCC and water temperature in the WTP effluent (N = 168) is shown in Fig 4A. However, careful inspection of the data showed that samples taken during the cooling period of each fall (August–January) and the warming period of each spring (February–July) of the two years displayed dissimilar correlations with temperature (Fig 4B and 4C), suggesting that temperature was not the only driving factor for the TCC changes in the WTP effluent. Possible secondary influencing factors for the TCC seasonal variations include abiotic factors. TCC (N = 176) was significantly and positively correlated to pH (p<0.001, r = 0.70), and inversely correlated with conductivity (p<0.001, r = -0.38) and concentration of assimilable organic carbon (AOC) (p<0.001, r = -0.52), the three parameters also displaying seasonal variations (S2 Fig; Fig 5A).

Water temperature in the WTP effluent was strongly and inversely correlated to AOC concentrations (p<0.001; r = -0.79; N = 168) (Fig 3), with AOC displaying an inverse seasonal trend compared to the water temperature and bacterial cell concentrations over the two-year period (Fig 5). The lowest AOC concentrations (3.5 μg Ac-C eq. L^-1^) were recorded at highest water temperatures and highest bacterial cell concentrations, and vice-versa, with highest AOC concentrations reaching 41.4 μg Ac-C eq. L^-1^ in winter (Fig 5). The high AOC and low TCC values in winter times in the WTP effluent can be attributed to the effect of low water temperature on bacterial activity in the biofilters (rapid sand filter and granular activated carbon filter) that would result in a low bacterial production and low AOC removal capacity [34,35]. The TCC in the WTP effluent was indeed fully controlled by the microbial dynamics in treatment steps subsequent to ozonation (which removes the majority of bacteria), namely rapid sand filtration and granular activated carbon filtration which both act as biofilters (S3 Fig). Addition of chlorine dioxide prior storage in the reservoir did not affect significantly the total bacterial cell concentration but did influence greatly bacterial viability, as the percentage of intact cells decreased from 86% after the GAC down to 14% after contact time with chlorine dioxide in the storage reservoir.

### Characterization of microbiological changes during water distribution

**Fig 6:**
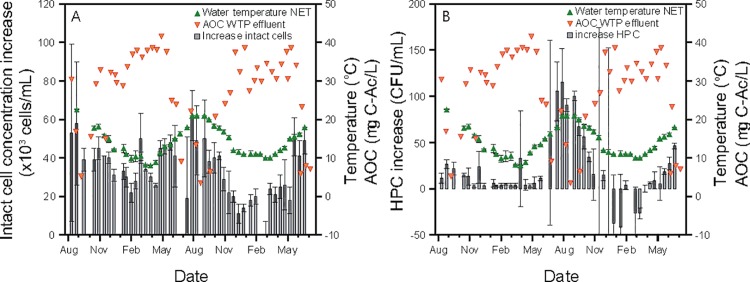
**Variations in time over two years (August 2012—June 2014) of the assimilable organic carbon (AOC) concentration in the treatment plant effluent (WTP) and the network (NET) water temperature, compared to (A) the intact cell concentration increase and (B) the HPC increase between the WTP effluent and the NET location.** Error bars indicate the standard deviation on four samples taken at the same location over a 2 h period.

**Fig 7:**
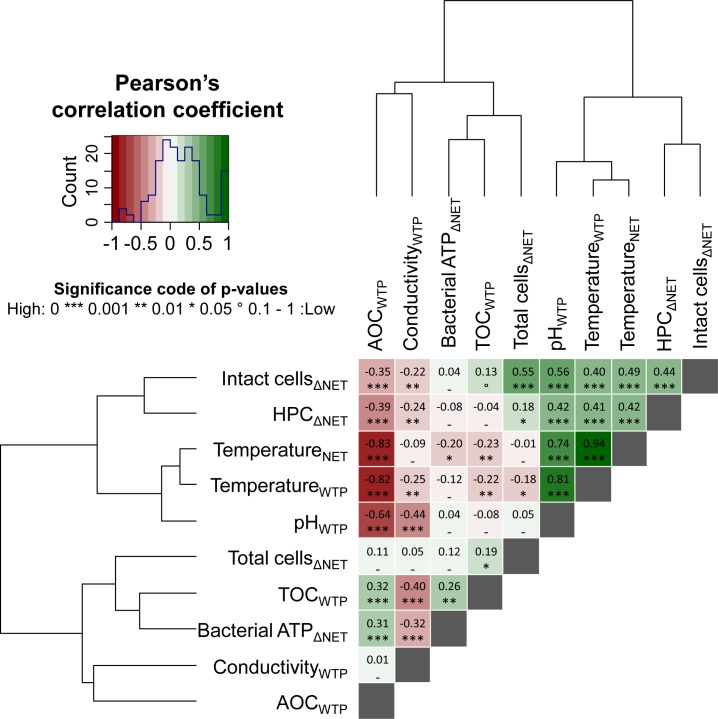
Heatmap of pair-wise Pearson’s correlation coefficients (*i*.*e*. linear trends) computed between the changes in microbial parameters (namely total cell concentration, intact cell concentration, bacterial ATP, and heterotrophic plate counts (HPC)) occurring in the distribution network (ΔNET) between the water treatment plant (WTP) effluent and the sampling location in the network (NET), and environmental parameters (namely temperature, pH, electrical conductivity, total organic carbon (TOC), and assimilable organic carbon (AOC)) in the WTP effluent and/or at the NET location (2-year dataset, 184 water samples in total). Hierarchical clustering using the Ward’s algorithm was first applied to reorder all parameters in clusters according to their correlation patterns as displayed by the dendrograms. The values and directions of the correlation coefficients are displayed according to the color key, *i*.*e*. positive correlations as green gradients from 0 to 1 and inverse correlations as red gradients from 0 to −1. Similar analysis of monotonic trends (*i*.*e*. Spearman’s rank-order correlations) between microbial and environmental parameters is available in S5 Fig. This approach is analogous to the one developed and used in [7] and [32].

The extent of change in microbial parameters between the WTP effluent and the NET varied over the year and followed a predictable trend similar to the water temperature but an unexpected inverse correlation to the AOC concentration in the water leaving the WTP. Fig 6 shows that the ICC increase between the two locations ranged between 11 × 10^3^ and 58 × 10^3^ cells mL^-1^, the highest increase being recorded in the warm periods. Similar observations were made for HPC, which increased significantly (up to 100 CFU mL^-1^) in the summer periods, and for *Aeromonas* counts (increase up to 427 CFU / 100 mL at warm temperatures). Water temperature was significantly and positively correlated to the change in intact cell concentration (p<0.001; r = 0.49; N = 168) and with the change in HPC (p<0.001; r = 0.42; N = 173) (Fig 7). The increase in *Aeromonas* counts also displayed a strong relationship with water temperature (S4 Fig). No or only small HPC increases (below 10 CFU mL^-1^) between the two sampling locations were recorded at low water temperatures, and the increase in bacterial ATP did not display seasonal trends. Overall, the strongest bacterial growth, as assessed by FCM, HPC, and *Aeromonas* analyses, was measured on the days with highest water temperatures, which corresponded to the lowest AOC concentrations in the WTP effluent. Although this result seems counter intuitive, it suggests that the AOC concentration in the water leaving the WTP was not the only bacterial growth-controlling factor within the distribution system at this specific NET sampling location. This argument is supported by a significant but inverse and weak linear correlation between the concentration of AOC in the WTP effluent and the increase in intact cells (p<0.001; r = -0.35; N = 168) and the increase in HPC (p<0.001; r = -0.39; N = 173) during water distribution (Fig 7).

## Discussion

Two-year monitoring of drinking water planktonic bacterial communities was performed at the water treatment plant (WTP) effluent and one location (NET) in a Dutch full-scale drinking water distribution system, operating without detectable residual disinfectant. Considerable seasonal variations in total (TCC) and intact (ICC) bacterial cell concentrations and bacterial ATP concentrations were detected in the WTP effluent, while no change was measured in HPC and *Aeromonas* (Figs 1 and 2). The TCC in the WTP effluent was governed by microbial dynamics in the biofiltration treatment steps, and clearly correlated to water temperature (Figs 3 and 4). Increases in microbial parameters during disinfectant-free water distribution were measurable, but were small compared to the variations observed in the WTP effluent (Figs 1 and 2). Bacterial growth in the distribution system was correlated with water temperature but not with the concentration of AOC in the WTP effluent (Figs 6 and 7).

### Seasonal variations in microbial community characteristics and microbial growth in a drinking water distribution system

The present study showed that large seasonal variations occurred in the microbial characteristics of drinking water (Figs 1 and 2) in the studied water treatment and distribution system. Large variations were observed in the WTP effluent in TCC, ICC and ATP concentrations, with bacterial cell concentrations being up to 5 times higher in the summer than in the winter period (Figs 1 and 2). Such seasonal variations were attributed to microbial processes occurring in biofilters, which are potentially affected by changes in the nutrient composition in the raw water (e.g. due to increased rainfalls or algal blooms in the case of surface waters) [36,37], or by changes in treatment efficiency (e.g. reduced biological activity in biofilters at low water temperatures) [34,35], and would need further investigation.

The extent of bacterial growth between the two locations was also shown to vary seasonally, and potential environmental growth-controlling conditions were investigated. Specific attention was given to water temperature and to assimilable organic carbon (AOC) concentration in the WTP effluent (Fig 6). Water temperature is indeed a major factor in the bacterial growth kinetics [38], whereas AOC is often depicted as the main microbial growth-controlling nutrient in drinking water [39–42]. The AOC assessment method applied in this study was based on the growth of two pure strains in a pasteurized water sample. Though this method only covers a fraction of all available organic compounds for growth in the water [43], the method has been consistently applied following the same procedure and thus provides a good indication for temporal changes in the available organic carbon pool. We showed in this way that AOC was varying seasonally with an opposite trend to water temperature and TCC (Fig 5), which was partially attributed to the treatment applied at this specific water work. Additionally, the results suggest that AOC concentration in the WTP effluent was not determining the bacterial growth at the studied network sampling location in this particular distribution system (Figs 6 and 7). In fact, the highest bacterial growth in the network was recorded at times where the lowest AOC concentrations were measured in the water leaving the WTP, which were below 10 μg Ac-C eq. L^-1^. The AOC value is commonly used to qualify a drinking water as biological stable in systems without detectable residual disinfectant [42,44]. Possible alternative controlling factors include inorganic nutrients such as phosphate, which was reported by the water utility to be always below detection limit and could not be further investigated. However, one cannot exclude the hypothesis that the available AOC in the water was not fully consumed at the NET sampling site, given the short residence time of approximately 2 days of the water until the NET sampling point. In addition, bacterial growth rates and yield on AOC in drinking water are likely influenced by water temperature [33,44], which would impact the AOC consumption kinetics in winter and in summer.

One should note that bacterial growth-limitations in a given distribution system are likely to change over a year in conjunction with the seasonal variations in microbial and chemical compositions of the treatment effluent and water temperature, such as displayed in this study. Pinto et al. [6] have recently shown that different environmental parameters such as pH and concentrations in ammonium, phosphate, sulphate and TOC are responsible for the changes in amplicon-sequencing profiles of bacterial community structures over a year. In distribution networks where residual disinfection is applied to control bacterial growth, the residual disinfection concentration is also an obvious parameter to consider. The disinfectant decay is likely to be faster at warmer water temperatures, higher bacterial cell concentrations, and higher organic carbon concentrations. This results in distribution areas with low residual concentrations of disinfectant at specific times of the year that can be subjected to increased bacterial growth [4,10,11,45].

### Unified monitoring strategy for the study of system-specific variations

Microbial dynamics in water treatment and distribution systems should be considered as site-dependent. The present study showed that large seasonal variations occurred in the microbial characteristics of drinking water (Figs 1 and 2) produced from surface water with an extensive treatment scheme including ozonation followed by biological filtration, and distributed with final disinfection but without detectable residual disinfectant. In comparison, no significant TCC variations were observed in the effluent of a Swiss large drinking water infrastructure with similar treatment steps and the following distribution system [25]. The different behaviour in microbial responses in the treatment effluent can be due to numerous factors such as different temperature variations within the treatment plant, or due to unique features associated with the raw water and the distribution characteristics (e.g. pipe material). It is recommended to apply a standard approach and unified methods, as detailed in Prest et al., 2016 [46], for direct comparison between individual water treatment and distribution systems, and to evaluate if the observation made in the present system can be generalized. Studies in literature are far from applying the same approach and important aspects detailed below are often left aside. The present study is a striking example that temporal dynamics can only be described by a long-term and high-frequency sampling strategy. A one-off or infrequent grab sampling approach would not have been representative for other periods of the year. However, temporal variations are rarely taken into account or discussed when conclusions are drawn from short-term or one-time studies.

Besides, it is critical to always include the treatment effluent in the sampling scheme when spatial and temporal dynamics are investigated. In the present study, spatial changes between the WTP and NET locations were minor, and water in the distribution network was mainly reflecting the water microbial characteristics leaving the treatment plant (Fig 1). Similar observations have been made in Dutch and Swiss facilities treating ground or surface water and distributing drinking water without detectable disinfectant residuals [12,19], in which bacterial cells concentrations in the range of 10^5^ cells mL^-1^ and ATP concentrations in the range of 12 ng L^-1^ remained stable during water distribution. The bacterial community composition analysed by pyrosequencing was also very similar in these treatment effluent and non-chlorinated distribution systems [12,24], as well as in an American network operating with residual disinfectants [6]. Studies focussing on water quality in the distribution system might draw erroneous conclusions in such cases with regards to the cause of temporal variations when the treatment effluent is not included in the sampling scheme.

### A combination of parameters for a complete description of bacterial community in water

Our study highlights that a combination of bioanalytical methods was essential for a complete description of the microbial dynamics in water, as suggested elsewhere [5,12,28]. FCM TCC were indicative for bacterial cells produced in biological filters (S3 Fig), independently from the effect of chlorine dioxide added thereafter. On the other hand, ICC provided insight on the disinfection effect of chlorine dioxide on the bacteria [47,48]. While 80 to 85% of the bacterial cells were intact after the biofiltration steps (S3 Fig), ICC represented only 27 ± 11% on average of the TCC in the water after chlorine dioxide addition and storage in the clear water reservoir, in which the disinfectant was fully consumed down to below the minimum detection limit (<0.001 mg L^-1^). Bacterial cell membrane integrity is, however, not a guaranty for cell viability. The latter can be assessed by bacterial ATP concentrations, which were below detection limit (1 ng L^-1^) on average over the two years in the WTP effluent. It was estimated that on average 15% of the intact cells in the WTP effluent were active, even after the addition of chlorine dioxide, by converting the bacterial ATP concentrations to active cell concentrations, using the conversion factor of 1.75 × 10^−10^ nmol ATP/cell proposed by Hammes and co-workers [18]. However, bacterial viability in the WTP effluent was not detected by the cultivation-based measurement of heterotrophic bacteria (i.e. cultivable HPC bacteria) and *Aeromonas*. The extremely low plate counts of both types of organisms in the WTP effluent over the two-year sampling period (Fig 2) can be attributed to the sensitivity to chlorine dioxide of *Aeromonas* [48] and cultivable HPC bacteria, that possibly enter a “viable but non culturable” state [49–51]. In the disinfectant-free distribution system, however, significant increase in *Aeromonas* counts (Fig 2C and S4 Fig) and in HPC counts in the case of warm water temperatures (above 15°C; Fig 6B) were indicative for bacterial growth. Overall, the study showed that the HPC and *Aeromonas* parameters are meaningful under conditions that notably lead to changes such as high temperatures and absence of residual disinfectant. However, the cultivation-based methods were less sensitive than the FCM and ATP methods, particularly in the presence of disinfectant residuals. Moreover, analytical results from HPC and *Aeromonas* measurements are only obtained after a minimum of two days after water sampling, which is significantly longer than the time required from FCM and ATP analysis (in the range of minutes). HPC and *Aeromonas* monitoring is nevertheless useful for comparison with temporal archives (feed-forward control), while rapid and sensitive measurements such as FCM and ATP ones are necessary for rapid corrective actions (feed-back measures).

In addition to these bacterial quantification methods, the value of 16S rRNA gene based fingerprinting (e.g. DGGE or T-RFLP) and amplicon sequencing methods (e.g 454-pyrosequencing or Illumina) has also previously been demonstrated for the study of microbial community composition and structure in water during treatment [6,21,24] and distribution [6,21,26,27].

### Long-term assessment of natural fluctuations in drinking water distribution systems

To reveal natural variations in drinking water quality for a specific treatment and distribution system, LeChevallier and co-workers [9] advised to collect large datasets of monitoring results for routine environmental parameters such as pH, temperature, free-chlorine residuals, and for coliforms, HPC, and AOC tests. In the present study, the approach has been extended to parameters such as ATP and FCM TCC and ICC, and large data sets (368 drinking water samples) were collected over a two-year period. The complete dataset clearly revealed that the considerable variations in TCC and ICC over the measuring period (Fig 1) were natural seasonal fluctuations for this particular water treatment and distribution system. The data can be used to estimate a range of predictable values for a defined period of time or water temperature (e.g. winter vs. summer; Fig 4C). Moreover, long-term measurements of TCC, ICC, HPC and *Aeromonas* showed that increased risk for uncontrolled bacterial growth during water distribution is expected at warm temperatures (Fig 6 and S4 Fig). In this framework, the value of numerical ecology methods was demonstrated for rationalization of large analytical datasets collected through high-frequency water monitoring, in order to highlight the main correlative trends between microbial, environmental, and engineering variables ([7,32], this study). The approach of long-term, high frequency monitoring and the definition of predictable ranges of values could be applied to any parameter of interest, and would be particularly useful for newly introduced parameters, such as FCM, that are not related to any guideline value and for which reference data are lacking. In this respect one could envision in future the use of continuous on-line microbial monitoring [7], as already routinely performed for several abiotic process parameters (e.g., pH, temperature, …).

## Conclusions

Two-year and high-frequency drinking water monitoring in a full-scale drinking water distribution system operated without residual disinfectant, using a combination of conventional and alternative microbial analytical methods led to the following key scientific conclusions, and recommendations for the practice:

■ Large seasonal variations occurred in bacterial cell concentrations and viability of the drinking water analysed at the effluent of the treatment facility. Assessment of temporal dynamics of microbial parameters is a key approach toward improved understanding of full-scale water systems.
■ Microbial characteristics of drinking water in the network were highly dependent on the characteristics of the water leaving the treatment facility; only minor changes occurred during distribution. Investigation of spatial microbial dynamics in distribution systems should always include detailed characterization of the treatment effluent.
■ Bacterial growth occurring during distribution up to the specific network location was not controlled by AOC concentrations in the treatment effluent, but was well correlated with water temperature. Likely not only one single parameter can be considered as controlling factor of microbial growth in distribution networks. Growth-controlling parameters are expected to change in time in a specific system. Future research should therefore specifically target mechanisms that drive microbial dynamics in distribution systems.
■ The collection of a large dataset by long-term and high-frequency monitoring of drinking water quality parameters sustained the assessment of natural fluctuations in microbial communities, as well as the prediction of microbial parameters based on water temperature and seasons.

## Supporting Information

S1 Fig Heatmap of pair-wise Spearman’s correlation coefficients (*i*.*e*. monotonic trends) computed between the microbial parameters (namely total cell concentration, intact cell concentration, bacterial ATP, and heterotrophic plate counts (HPC)), and the environmental parameters (namely temperature, pH, electrical conductivity, total organic carbon (TOC), and assimilable organic carbon (AOC)) measured in the effluent of the water treatment plant (2-year dataset, 184 water samples in total). Hierarchical clustering using the Ward’s algorithm was first applied to reorder all parameters in clusters according to their correlation patterns as displayed by the dendrograms. The values and directions of the correlation coefficients are displayed according to the color key, *i*.*e*. positive correlations as green gradients from 0 to 1 and inverse correlations as red gradients from 0 to −1. This approach is analogous to the one developed and used in [7] and [32].
(TIF)

S2 Fig **Temporal variations over two years (August 2012—June 2014) in the (A) concentration of total organic carbon (TOC), (B) pH, and (C) electrical conductivity of the drinking water at the outlet of the water treatment plant (WTP) and at one location in the water distribution network (NET).** Error bars indicate the standard deviation on four samples taken at the same location over a 2 h period.
(TIF)

S3 Fig Flow cytometry analysis of water samples taken after each treatment step (sampling on a single day at the water treatment plant; November 2012). (A) Total, intact and damaged bacterial cell concentrations, (B) cell concentrations after the ozonation treatment step (enlarged from A). Legend: WTP: water treatment plant; coag/sed: coagulation and sedimentation; RSF: rapid sand filtration; GAC: granular active carbon filtration; ClO2: chlorine dioxide addition.
(TIF)

S4 Fig Relationship between the temperature of drinking water at the outlet of the water treatment plant (WTP) and the increase in the counts of *Aeromonas*, between samples taken at the WTP effluent and at the sampling location in the water distribution network during the two year investigation period (August 2012 –June 2014). (TIF)

S5 Fig Heatmap of pair-wise Spearman’s correlation coefficients (*i*.*e*. monotonic trends) computed between the changes in microbial parameters (namely total cell concentration, intact cell concentration, bacterial ATP, and heterotrophic plate counts (HPC)) occurring in the distribution network (ΔNET) between the water treatment plant (WTP) effluent and the sampling location in the network (NET), and environmental parameters (namely temperature, pH, electrical conductivity, total organic carbon (TOC), and assimilable organic carbon (AOC)) in the WTP effluent and/or at the NET location (2-year dataset, 184 water samples in total). Hierarchical clustering using the Ward’s algorithm was first applied to reorder all parameters in clusters according to their correlation patterns as displayed by the dendrograms. The values and directions of the correlation coefficients are displayed according to the color key, *i*.*e*. positive correlations as green gradients from 0 to 1 and inverse correlations as red gradients from 0 to −1. This approach is analogous to the one developed and used in [7] and [32].
(TIF)

S1 Text Analysis of flow cytometric fluorescence fingerprints–methods and results. (DOCX)

S2 Text Multivariate numerical analyses applied to the 2 year datasets collected from the effluent of the water treatment plant (WTP). (DOCX)

## Abbreviations

AOC: assimilable organic carbon
ATP: adenosine-tri-phosphate
CFU: colony forming unit
FCM: flow cytometry / flow cytometer
HPC: heterotrophic plate counts
ICC: intact bacterial cell concentration
NET: distribution network
TCC: total bacterial cell concentration
TOC: total organic carbon
WTP: water treatment plant
