# Supplementary figures and images for: Long-Term Bacterial Dynamics in a Full-Scale Drinking Water Distribution System
Source: PLoS One. 2016 Oct 28;11(10):e0164445. doi: 10.1371/journal.pone.0164445 (PMC5085035; doi:10.1371/journal.pone.0164445)

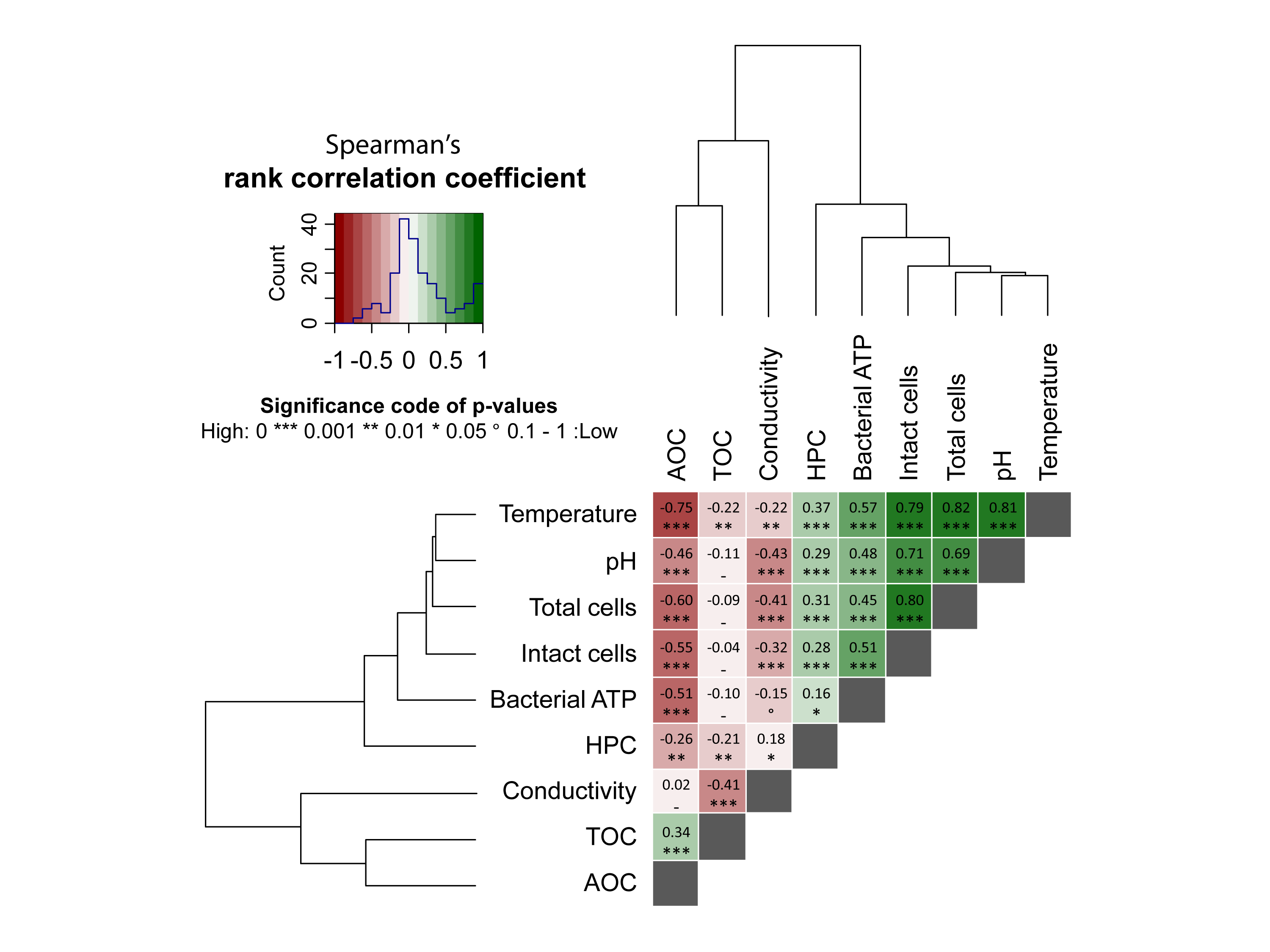

Supplement: S1 Fig — Hierarchical clustering using the Ward’s algorithm was first applied to reorder all parameters in clusters according to their correlation patterns as displayed by the dendrograms. The values and directions of the correlation coefficients are displayed according to the color key, i.e. positive correlations as green gradients from 0 to 1 and inverse correlations as red gradients from 0 to −1. This approach is analogous to the one developed and used in [7] and [32]. (TIF) [file pone.0164445.s001.tif]

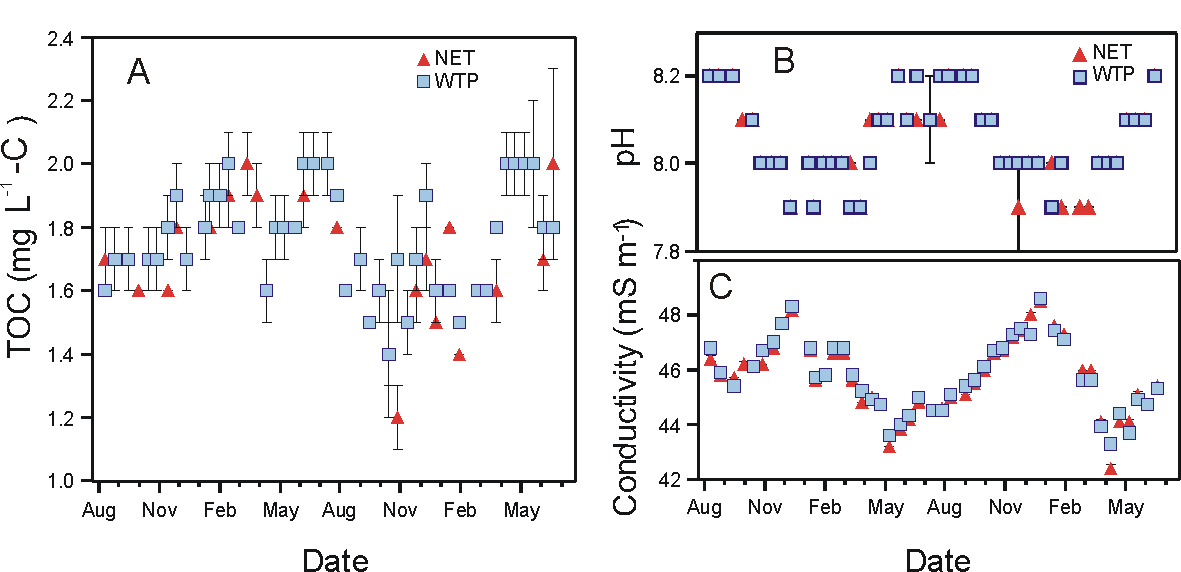

Supplement: S2 Fig — Temporal variations over two years (August 2012—June 2014) in the (A) concentration of total organic carbon (TOC), (B) pH, and (C) electrical conductivity of the drinking water at the outlet of the water treatment plant (WTP) and at one location in the water distribution network (NET). Error bars indicate the standard deviation on four samples taken at the same location over a 2 h period. (TIF) [file pone.0164445.s002.tif]

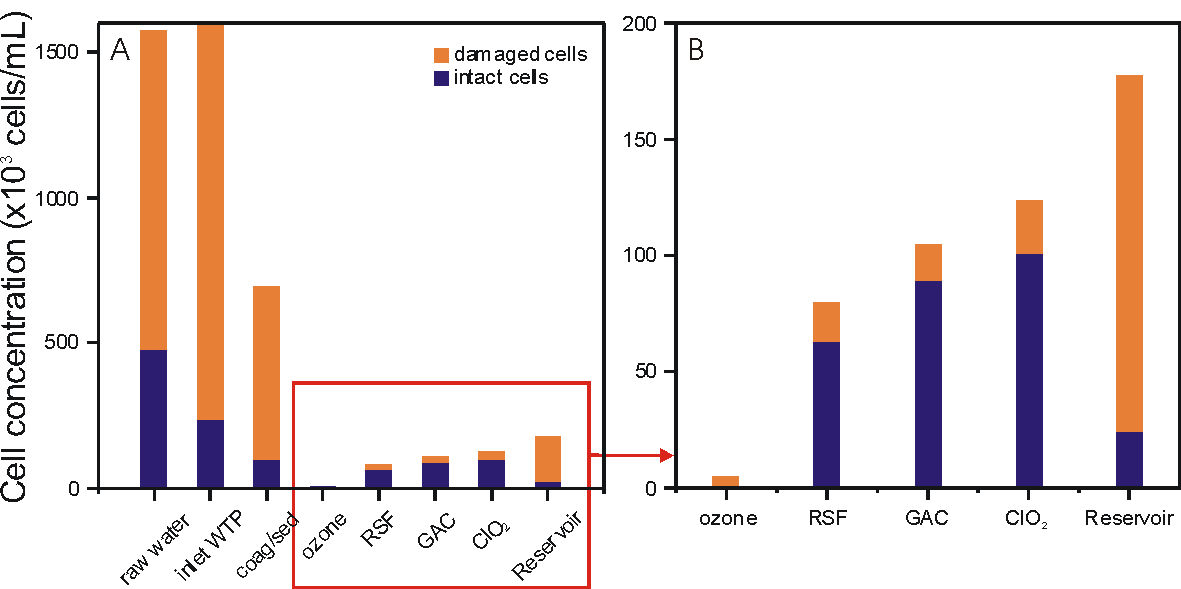

Supplement: S3 Fig — (A) Total, intact and damaged bacterial cell concentrations, (B) cell concentrations after the ozonation treatment step (enlarged from A). Legend: WTP: water treatment plant; coag/sed: coagulation and sedimentation; RSF: rapid sand filtration; GAC: granular active carbon filtration; ClO2: chlorine dioxide addition. (TIF) [file pone.0164445.s003.tif]

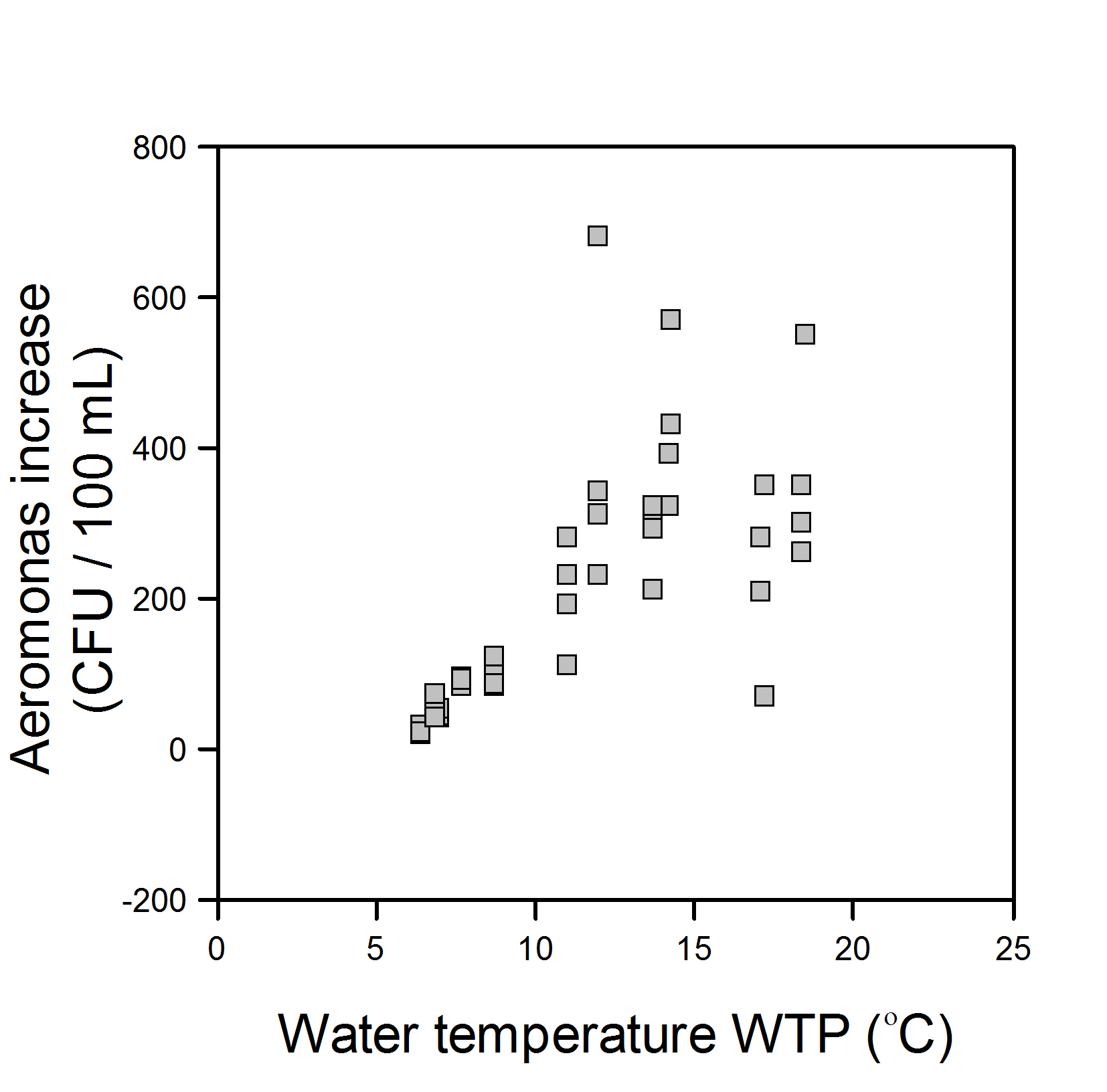

Supplement: S4 Fig — (TIF) [file pone.0164445.s004.tif]

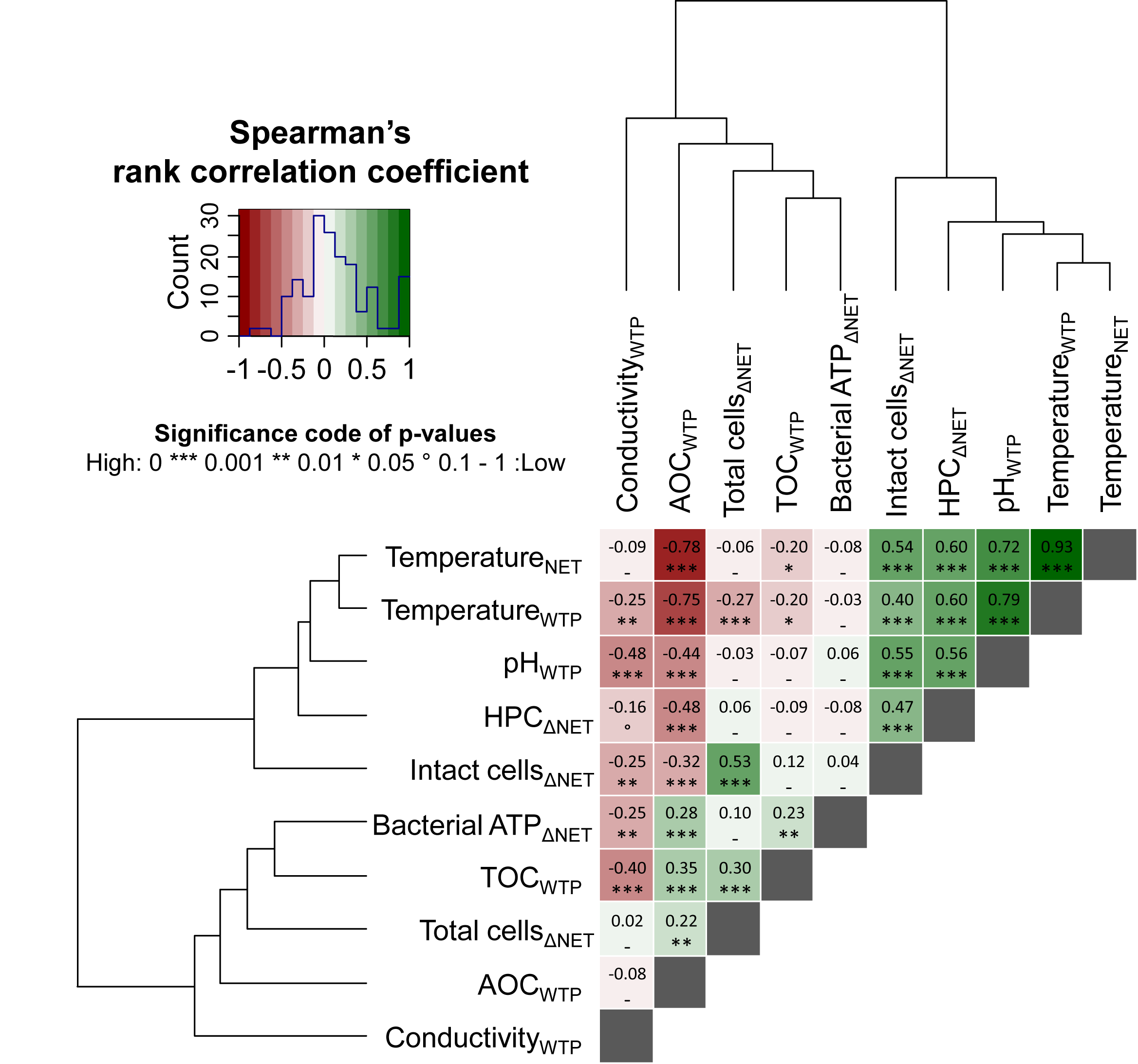

Supplement: S5 Fig — Hierarchical clustering using the Ward’s algorithm was first applied to reorder all parameters in clusters according to their correlation patterns as displayed by the dendrograms. The values and directions of the correlation coefficients are displayed according to the color key, i.e. positive correlations as green gradients from 0 to 1 and inverse correlations as red gradients from 0 to −1. This approach is analogous to the one developed and used in [7] and [32]. (TIF) [file pone.0164445.s005.tif]
