# Supplementary material for: Long-Term Bacterial Dynamics in a Full-Scale Drinking Water Distribution System
Source: PLoS One. 2016 Oct 28;11(10):e0164445. doi: 10.1371/journal.pone.0164445 (PMC5085035; doi:10.1371/journal.pone.0164445)
Supplement: S1 Text — (DOCX) [file pone.0164445.s006.docx]

**S1 text. Analysis of flow cytometric fluorescence fingerprints**

Fluorescence fingerprints of drinking water samples typically display two clusters differentiated by fluorescence intensity, corresponding to bacteria with low (LNA) and high (HNA) nucleic acid content. LNA and HNA clusters were separated using fixed electronic gates and quantification and straightforward comparison of fingerprints from different water samples was made using the percentage of HNA cells, compared to total cells (Prest et al., 2013).

Flow cytometric (FCM) fluorescence fingerprints are indicative of the bacterial community composition in drinking water samples (Prest et al., 2014). The fingerprints obtained based on intact cell enumeration conducted using staining with a mixture of SYBR Green I and propidium iodide were examined, but neither revealed variations in time in the percentage of HNA cells at both locations nor displayed clear seasonal trends (Figure S1a). However, a clear shift in bacterial community composition occurred during water distribution, with a consistent and significant (P<0.0001) increase in the percentage of high nucleic acid (HNA) bacterial cells between the two locations (increase from 41.5 to 50.9 % on average). The fingerprints obtained from total cell enumeration, conducted using staining with SYBR Green I only, did not display seasonal variations at the WTP effluent nor in the network (Figure S1b). Significant (P=0.0006 based on a paired t-test) but very limited change in the percentage of HNA cells (from 40.3 to 30.9 % on average over the two years) was revealed between the two locations. This can be due to measurement sensitivity, as the water samples contained a large amount of damaged bacterial cells (70 % on average), probably hampering the detection of small changes occurring within the intact fraction of bacterial cells. The temporal and spatial variations in bacterial community composition will be further examined in depth in a separate self-contained article on high-throughput investigation of microbial community dynamics using 16S rRNA gene-based amplicon-sequencing analysis.

**
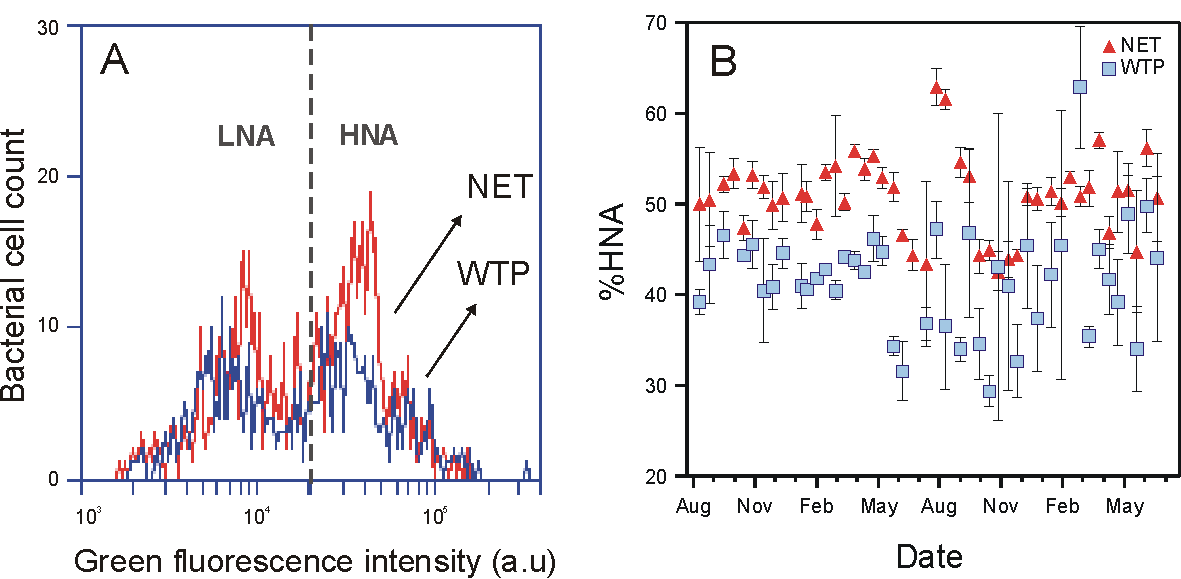
**

S1a Fig. Bacterial community analysis using flow cytometric fingerprints based on intact cell measurements (bacterial staining with SYBR Green I and propidium iodide). (A) Comparison of unprocessed fingerprints (green fluorescence distribution) of drinking water samples taken at the water treatment plant (WTP) effluent and at one location in the network (NET) on the same day. (B) Variations in time over two years (August 2012 - June 2014) of percentage of high nucleic acid bacterial cells (HNA) at the two locations. Error bars indicate the standard deviation on four samples taken at the same location over a 2 h period.

**
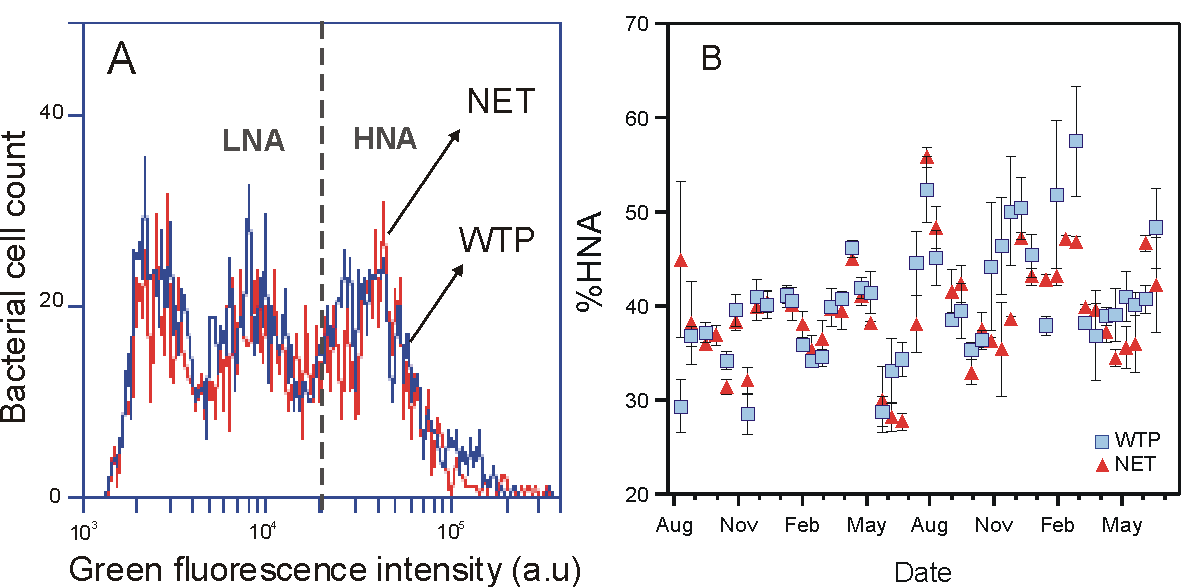
**

S1b Fig. Bacterial community analysis using flow cytometry (FCM) fingerprints based on total cell measurements (bacterial staining with SYBR Green I only). (A) Comparison of unprocessed fingerprints (green fluorescence distribution) of drinking water samples collected at the water treatment plant (WTP) effluent and in the water distribution network (NET) on the same day. (B) Temporal variations over two years (August 2012 - June 2014) of the percentage of high nucleic acid bacterial cells (HNA) at the two sampling locations. Error bars indicate the standard deviation on four samples taken at the same location over a 2 h period.
