# Supplementary material for: Long-Term Bacterial Dynamics in a Full-Scale Drinking Water Distribution System
Source: PLoS One. 2016 Oct 28;11(10):e0164445. doi: 10.1371/journal.pone.0164445 (PMC5085035; doi:10.1371/journal.pone.0164445)
Supplement: S2 Text — (DOCX) [file pone.0164445.s007.docx]

**S2 text. Multivariate numerical analysis applied to the 2 year datasets collected from the effluent of the water treatment plant (WTP)**

**S2a Fig. Panel plot of dynamics in individual measured variables over time in order to delineate global trends (concatenated 2 year WTP datasets, 184 water samples in total).**

**S2b Fig. Pair-wise x-y plots between all measured variables for preliminary visual observation of apparent linear and monotonic correlations (concatenated 2 year WTP datasets, 184 water samples in total). The meaning of abbreviations are given in Table S2.**

S2 Table. Overview of abbreviations in use in Figures S2b to S2e.

| **Total cells** | Total cell concentration, as determined with flow cytometry following bacterial staining with Sybr Green I only |
| --- | --- |
| **Intact cells** | Intact cell concentration, as determined with flow cytometry following bacterial staining with a mixture of Sybr Green I and propidium iodide |
| **X.intact** | Percentage of intact cells compared to total cells |
| **NormFl1.SG** | Normalized green fluorescence as determined with flow cytometry following bacterial staining with Sybr Green I only (Prest et al., 2014) |
| **X.HNA.SG** | Percentage of high nucleic acid cells compared to total cells, as determined with flow cytometry following bacterial staining with Sybr Green I only (Prest et al., 2014) |
| **NormFl1.SGPI** | Normalized green fluorescence as determined with flow cytometry following bacterial staining with a mixture of Sybr Green I and propidium iodide (Prest et al., 2014) |
| **X.HNA.SGPI** | Percentage of high nucleic acid cells compared to total cells, as determined with flow cytometry following bacterial staining with a mixture of Sybr Green I and propidium iodide (Prest et al., 2014) |
| **Bact.ATP** | Bacterial ATP |
| **HPC** | Heterotrophic plate count |
| **TOC** | Total organic carbon concentration |
| **AOC** | Assimilable organic carbon concentration |
| **WTP** | Refers to samples taken at the water treatment plant effluent |
| **NET** | Refers to samples taken at the distribution network sampling location |
| **ΔNET** | Refers to changes measured between water treatment plant effluent and distribution network sampling location |

**S2c Fig. Self-contained Pearson’s correlation matrix used to delineate linear correlative trends between variables (concatenated 2 year WTP datasets, 184 water samples in total). The upper section of the matrix displays Pearson’s correlation coefficient, that indicate positive (red) or negative (blue) correlative trends.**

**S2d Fig. Graphing and hierarchical clustering using the Ward’s algorithm of measured variables displaying analogous correlation patterns with all other variables. The heat map provides a straightforward representation of the gradients in positive and inverse Pearson’s correlations (*i.e.* linear trends) between variables toward rapid identification of major correlative trends (concatenated 2 year WTP datasets).**

**S2e Fig. Comparison of the half heat maps of Pearson’s correlation (*i.e.* linear trends) and Spearman’s rank-order correlations (*i.e.* monotonic trends) between measured variables originating from the datasets collected from the effluent of the water treatment plant (WTP, panel A1-2) and from the changes in the water distribution network (ΔNET, panel B1-2), over the 2-year water analysis campaign. In the latter dataset, the absolute value of the water temperature in the network (temperature_NET_) was considered.**
